# Sociodemographic and Clinical Determinants of Psychiatric Hospitalization in Northern Greece: A descriptive study

**DOI:** 10.1192/j.eurpsy.2024.1191

**Published:** 2024-08-27

**Authors:** G. N. Porfyri, P. Papadopoulou, M. V. Karakasi, A. Vlachaki

**Affiliations:** ^1^Psychiatry Department, General Hospital of Papanikolaou, Thessaloniki; ^2^Psychiatry Department, University General Hospital of Alexandroupolis, Alexandroupolis, Greece

## Abstract

**Introduction:**

According to data, psychiatric re-admissions rates vary from 10%-80%, while they negatively affect the patients’ quality of life and life expectancy. The limitation of multiple psychiatric hospitalizations represents a clinical challenge for all mental health professionals.

**Objectives:**

To investigate risk factors of hospitalization in a sample of psychiatric patients in Northern Greece.

**Methods:**

1,633 records of psychiatry inpatients were examined retrospectively throughout the 10-year records of the Psychiatry Department of Papanikolaou General Hospital in northern Greece. The research was conducted between 2013 and August 2023. The sample was divided into subgroups according to gender, diagnosis, and year of hospitalization. A bivariate analysis was performed to examine relationships between the examined variables: (a.) place of residence; (b.) age; (c.) type of admission; (c.) hospitalization duration; (d.) number of lifetime hospitalizations; (e.) lifetime prosecutor’s orders for coercive examination; and (f.) lifetime suicide attempts.

**Results:**

A fairly equivalent number of males and females was included in the study (M: 874; F: 759).The mean age of the sample was 44.7 years with males being younger than females (males 43.23; females 46.39). Males residing out of the co-capital as well as females residing within the co-capital of Greece, Thessaloniki, disclosed higher odds of being hospitalized (p<0.03). Coercive hospitalizations represented 47% of cases, bore the highest duration (20.7 days), and involved the youngest patients. Coercively hospitalized male patients outnumbered their female counterparts (p<0.001). Voluntary urgent hospitalizations duration was estimated at 17.04 days, followed by outpatient admissions (12.64 days) and transfers from other clinics (11.35 days). 37% of patients experienced psychosis while 35% experienced affective disorders. Males were more affected by psychosis (Odds Ratio: 1.35; p<0.001). Females were more liable to affective disorders (OR: 1.78; p<0.001). 7% of the sample had committed suicide attempts, with single suicide attempts being ten times higher than multiple suicide attempts (p<0.001). Females were more than twice as likely as males to commit a suicide attempt (p<0.001). Females tended more to be hospitalized self-willingly (p=0.0015) and to voluntarily terminate hospitalizations prematurely (p=0.0014). Patients with a single hospitalization were seven-fold compared to those with multiple hospitalizations (p<0.001). The average lifetime hospitalization number for a patient was one hospitalization, while the average for a patient with previous hospitalizations was three.

**Conclusions:**

Being in position to identify the patients in high-risk for hospitalization -as well as for suicide attempt- the clinician can proceed to initiatives such as treatment modifications or further involving the patient’s family.

**Disclosure of Interest:**

None Declared

